# Syndrome of inappropriate antidiuresis secondary to multiple myeloma: a case report and literature review

**DOI:** 10.3389/fonc.2026.1697444

**Published:** 2026-01-29

**Authors:** Linling Fan, Ting Ding, Liya Mao, Qian Wang, Hongying Ye, Yiming Li, Shuo Zhang, Lijin Ji

**Affiliations:** 1 Department of Endocrinology and Metabolism, Huashan Hospital, Fudan University, Shanghai, China; 2 Department of Endocrinology and Metabolism, Yiyang Central Hospital, Hunan, Yiyang, China; 3 Department of Hematology, Huashan Hospital, Fudan University, Shanghai, China

**Keywords:** anti-myeloma therapy, hyponatremia, interleukin-6, multiple myeloma, reset osmostat, SiaD

## Abstract

The syndrome of inappropriate antidiuresis (SIAD) has been associated with various diseases. However, to our knowledge, only a few studies have reported multiple myeloma (MM) as a potential cause of SIAD. We report a case where hyponatremia served as the primary clinical manifestation, diagnosed with SIAD and subsequently confirmed as multiple myeloma. Following anti-myeloma therapy, the hyponatremia was successfully corrected upon achieving a hematologic response classified as Very Good Partial Response (VGPR). This case contributes to an uncommon etiology of SIAD, highlighting the potential association between SIAD and multiple myeloma, and underscores the need for further research into this connection.

## Introduction

The syndrome of inappropriate antidiuresis (SIAD) is the predominant cause of hypotonic hyponatremia, resulting from the non-osmotic release of arginine vasopressin (AVP, also known as ADH), which acts on the renal V2 receptors to promote water retention. The causes of SIAD include different types of solid organ tumors, medications, pulmonary conditions, disorders of the central nervous system, postoperative state, severe nausea, stress, etc (1). However, in some cases, the underlying cause remains undetermined. Few studies reported hematological malignancies as the potential cause of SIAD. To date, multiple myeloma (MM) has not been widely recognized as a potential cause of SIAD, though there were only three cases reported the correlation between MM and SIAD (2–4). In this case, we report another case of MM presenting hyponatremia as the primary clinical manifestation.

Our objective is to explore the potential relationship between MM and SIAD to improve clinical diagnosis and deepen understanding of SIAD’s underlying causes.

## Case report

A 78-year-old Chinese man was admitted to our department due to recurrent lower limbs weakness for one year in September 2023. He had experienced lower limbs weakness since September 2022. The patient had a documented decreased blood sodium level of 125 mmol/L during the hospitalization for myocardial infarction in April 2022. The patient received intermittent oral sodium supplementation at local hospitals. At the time of discharge, his sodium level had returned to 138 mmol/L. However, after discharge, he did not continue sodium supplementation or monitor his serum sodium levels and his weakness progressively worsened, along with difficulty walking. His blood sodium level dropped to a nadir of 120 mmol/L, prompting referral to our department in 2023.

Past Medical History: The patient had a 5-year history of hypertension treated with Candesartan, 8 years of hyperlipidemia, and 1 year of coronary heart disease, all well-controlled. He had been diagnosed with hypothyroidism for 4 years and was taking levothyroxine 75 μg daily, with normal recent thyroid function. Additionally, he had a history of leukopenia for nearly 1 year, treated with Leucogen tablets.

Physical examination: Vital signs: Temperature (T): 36.7 °C, Pulse (P): 73 beats per minute, Respiratory rate (R): 18 breaths per minute, Blood pressure (BP): 125/78 mmHg, Height: 169 cm, Weight: 71 kg, Body Mass Index (BMI): 24.86 kg/m². The patient’s skin and mucous membranes showed normal turgor, without dryness or edema; cardiac auscultation revealed normal heart sounds without distant tones, a normal heart rate, and regular rhythm; daily urine output was 2–3 liters. Muscle strength was normal, muscle tone was normal, and deep tendon reflexes were normal. No pathological reflexes were elicited. The patient exhibited an unsteady gait. Otherwise, the physical examination revealed no abnormalities.

Laboratory tests: sodium 123 mmol/L (normal range: 135–145 mmol/L), plasma osmolality of 257mOsm/kg per H_2_O (normal range: 280-300mOsm/kg per H_2_O), urine sodium 121 mmol/L (341 mmol/24 h), urine osmolarity 390mOsm/kg per H_2_O. Thyroid and adrenal cortex function was normal. Blood glucose and lipids levels were within the normal range. N-terminal pro-B-type natriuretic peptide (proBNP) levels were within the reference interval. The patient exhibited normal liver and kidney function, with no history of diuretic use. The patient’s other laboratory values were as follows: serum uric acid 0.2 mmol/L (<4 mg/dL), fractional excretion of sodium 1.28% (>0.5%), fractional excretion of urea 40.99%, and fractional excretion of uric acid 8.6%. The patient’s serum potassium and acid-base status were within normal limits. Hypotonic hyponatremia was confirmed with a diagnosis of SIAD. Fluid restriction and daily oral tolvaptan 3.75mg (Otsuka Co., Ltd, Lin-an, Zhejiang, China) corrected the sodium level.

We then investigated the etiology of SIAD. The patient had no history of brain trauma, pneumonia, or drug abuse. A lung CT scan showed scattered solid nodules, likely benign proliferative foci, with a 7×4.8 mm ground-glass opacity nodule in the apicoposterior segment of the left upper lobe. Gastroscopy revealed chronic colitis and fundic gland polyps. Brain MRI showed multiple lacunar infarcts, white matter degeneration, and brain atrophy. An 18F-fluorodeoxyglucose positron emission tomography/CT (18F-FDG PET/CT) scan revealed diffusely increased FDG uptake in the bone marrow, without significant increases elsewhere, including the brain. Tumor markers, inflammatory markers, and immune-related indicators were unremarkable.

**Figure 1:**
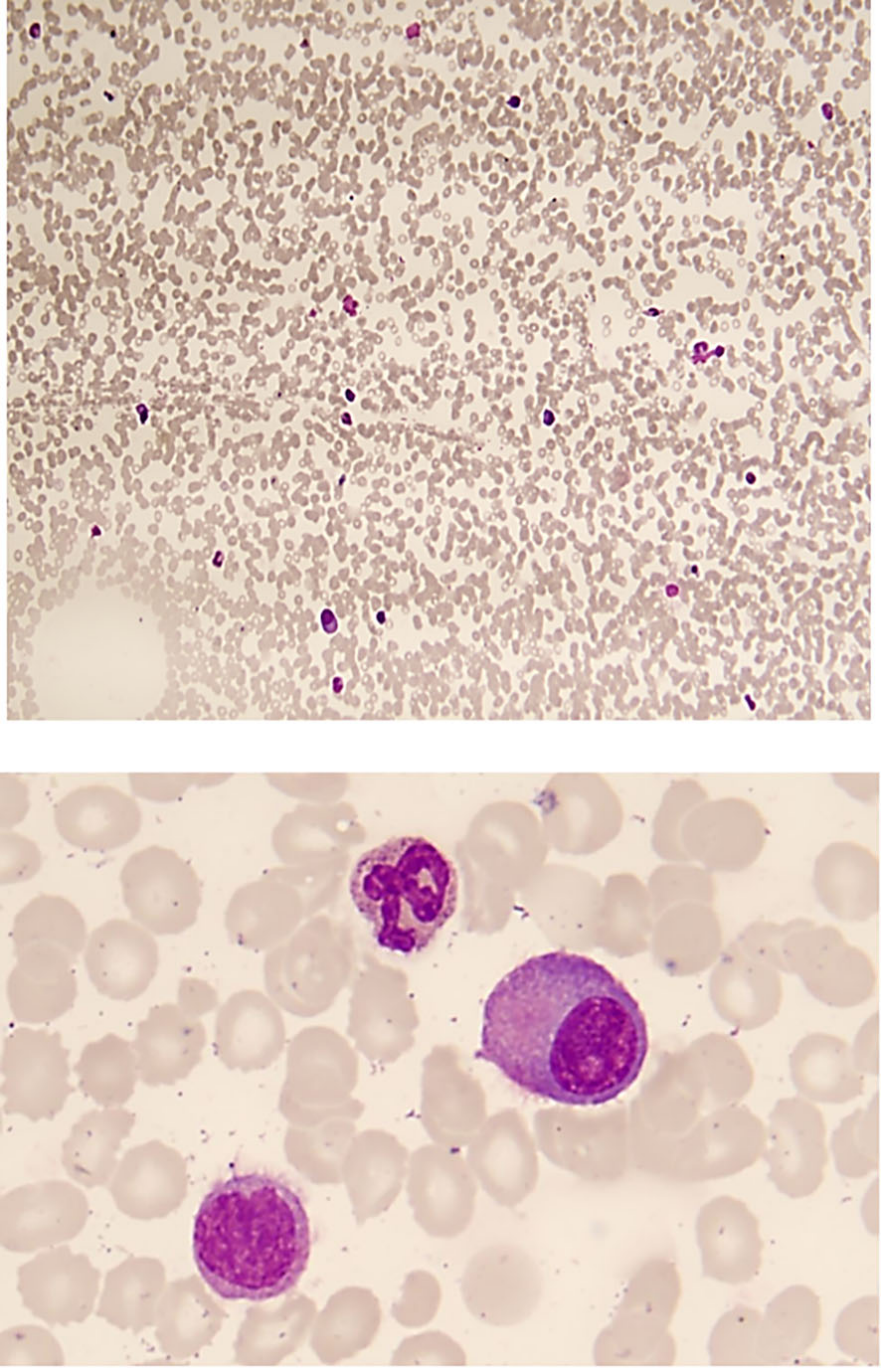
Examination of marrow smear reveals hypoplastic hematopoiesis, with prominent effects on the erythroid and megakaryocytic lineages, and no bone marrow particles are identified. The proportion of primitive plasma cells is abnormally elevated to 15.5% in the specimen, indicating: Multiple Myeloma.

White blood cells (WBC) 3.12*10^9/L, neutrophil 1.63*10^9/L, hemoglobin 120 g/L, albumin 45 g/L, globulin 30 g/L, creatinine 54 mmol/L, immunoglobulin (Ig) G was 15 g/L (normal range: 8.6-17.4 g/L) with suppressed levels of IgM 0.19 g/L (normal range: 0.35-2.20 g/L) and IgA 0.26 g/L (normal range: 1-4.2 g/L). Protein electrophoresis showed a monoclonal peak, the M component was 22.68%. Immunoelectrophoresis of serum proteins showed an IgG lambda M-protein. Blood light chain λ was 4.80 g/L (normal range: 0.9-2.1g/L), κ was 0.25 g/L (normal range: 1.7-3.7g/L), and the κ/λ ratio was 0.05 (normal range: 1.35-2.65). Urinary light chain λ was 28.10 mg/L (normal range: <4.1 mg/L), and urinary κ light chain was less than 7.16 mg/L (normal range: <7.5mg/L). Blood free light chain (FLC) λ was 471 mg/L (normal range: 8.3-27mg/L), κ was 8 mg/L (normal range: 6.7-22.4mg/L), and the κ/λ ratio was 0.02 (normal range:0.31-1.56). Vascular endothelial growth factor (VEGF) was 132.82 pg/ml (normal range 0-142pg/ml). Bone marrow flow cytometry analysis indicated the presence of abnormal plasma cells accounting for 7.54% of nucleated cells with strongly expressing CD_38_, CD_138_, cytoplasmic IgG and cytoplasmic λ. Examination of marrow smear presented an abnormal increase in the proportion of primitive plasma cells to 15.5% (**Figure 1**). The hematologist consultation diagnosed the patients with IgG-λ type of MM, stage IA (Durie-Salmon Staging System) (5).

**Figure 2:**
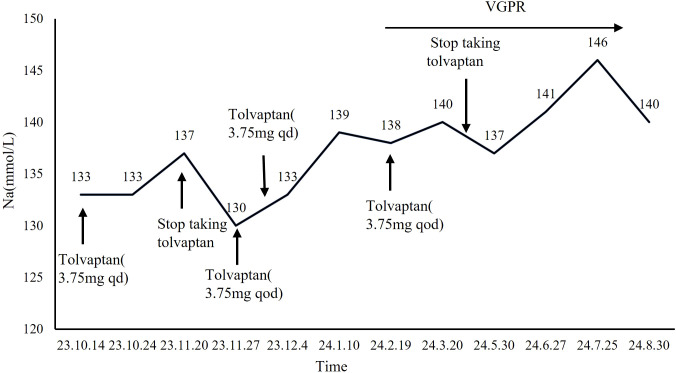
Serum sodium levels after treatment of MM.

The patient was treated in the hematology department with an anti-myeloma regimen consisting of bortezomib, lenalidomide, and dexamethasone (BRD) for 11 cycles. After 5 cycles, the patient achieved a hematologic response of Very Good Partial Response (VGPR). The Tolvaptan dose was gradually reduced and discontinued in April 2024 after 5 cycles of the BRD regimen (**Figure 2**). During follow-up, his serum sodium levels remained within the normal range of 137–146 mmol/L, and SIAD resolved following treatment for MM.

## Discussion

In this case, we report another case of MM presenting hyponatremia as the primary clinical manifestation. However, it is essential to consider the possibility that MM may lead to pseudohyponatremia due to the excessive secretion of monoclonal proteins (e.g., M proteins). Considering the low plasma osmolality and high urine osmolality with euvolemic hyponatremia, we have excluded the possibility of pseudohyponatremia. The diagnosis of SIADH was primarily entertained as further fluid restriction and tolvaptan administration corrected the hyponatremia (6).

The common causes of SIAD include solid organ tumors, medications, pulmonary conditions, central nervous system disorders, postoperative states, severe nausea, stress, and etc. Hematological malignancies were not common causes of SIAD. However, several cases reported MM as a potential cause of SIAD. The earliest case, reported in 1983, described a patient with IgG λ-type MM who likely developed SIAD-induced hyponatremia; unfortunately, the patient died before any further treatment could be administered and an autopsy was not subsequently performed (2). The second case, involving IgA λ-type MM with secondary SIAD, was treated with dexamethasone and bortezomib, resulting in complete resolution of the patient’s hyponatremia and anemia (3). The most recent case in 2020 involved SIAD associated with IgG κ-type MM, and the patient’s condition improved after the disease went into complete remission (**Table 1**) (4). Of note, although bortezomib-based anti-MM therapy may lead to hyponatremia, the introduction of bortezomib to our patients’ regimen did not lead to fluctuations in the plasma sodium concentration (7).

**Table 1:** Summary of cases about SIAD induced by multiple myeloma.

Publication date	Journal	Sex	age	MM type	Treatment	Prognosis
1983 [Nanji AA et al. (2)]	Southern Medical Journal	woman	71	IgG λ	No	Died
2011 [Abraham A et al (3)]	Science of Medicine	woman	61	IgA λ	dexamethasone and bortezomib	hyponatremia corrected after one cycle of chemotherapy and palliative care later for MM
2020 [Tang R et al (4)]	Chinese Medical Journal	man	60	IgG κ	methylprednisolone and bortezomib	hyponatremia corrected after two cycles of chemotherapy and MM CR for two years

In our case, the patient presented with hyponatremia secondary to SIAD as the primary clinical manifestation, alongside a confirmed diagnosis of MM. After receiving anti-myeloma therapy, the patient achieved VGPR, and his hyponatremia was successfully corrected. Although there is no direct evidence that multiple myeloma cells secrete ADH, a proposed mechanism suggests that increased interleukin-6 (IL-6) production by myeloma cells may stimulate ADH secretion (8–10). In our patient, elevated IL-6 levels (6.7pg/ml) were detected at diagnosis, supporting this hypothesis. Future studies could involve immunohistochemical staining for ADH in myeloma cells to confirm whether they directly cause inappropriate ADH secretion or indirectly mediated by IL-6. Notably, reset osmostat is an important differential diagnosis in cases suggestive of SIAD. However, for this patient, several clinical observations argue against it: sodium levels normalized with fluid restriction and supplementation, responded gradually to tolvaptan without overcorrection, and remained within the normal range following MM treatment without ongoing medication. However, the absence of a water-diluting test to definitively exclude this condition remains a study limitation.

In conclusion, we present another case of SIAD secondary to multiple myeloma, contributing to the understanding of the etiology of SIAD and highlighting the need for further research into this association.

## Data Availability

The datasets presented in this study can be found in online repositories. The names of the repository/repositories and accession number(s) can be found below: The data that support the findings of this study are available from the corresponding author upon reasonable request.
